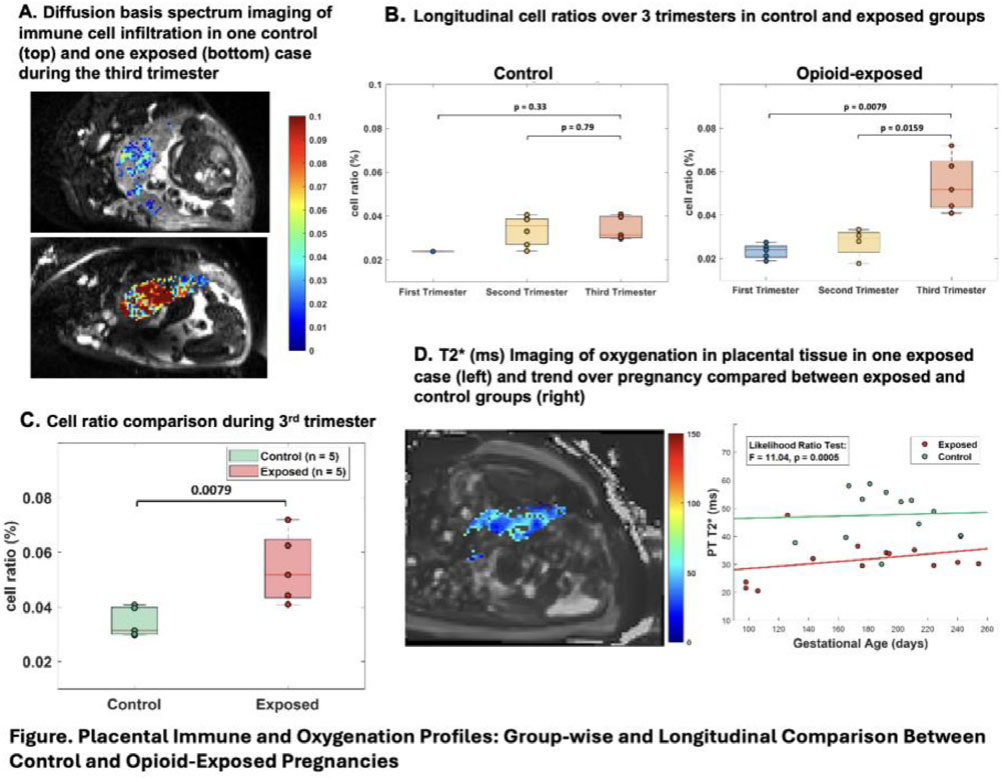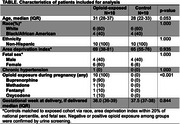# Oral Concurrent Session 1 – Medical and Surgical Complications of Pregnancy: Wednesday, February 11, 2026 1:00 PM – 3:30 PM

**DOI:** 10.1002/pmf2.70167

**Published:** 2026-02-09

**1:00 PM – 1:15 PM**

## 9 | Comparative Effectiveness of Two Enhanced Prenatal Care Models on Mental Health: Randomized Trial

Miriam Kuppermann^1^; Jennifer N. Felder^2^; Daisy León‐Martínez^3^; Deborah Karasek^4^; Venise Curry^5^; Kristin Carraway^5^; Bethany Simard^6^; Kimberly Coleman‐Phox^7^; Brittany D. Chambers Butcher^8^; Patience A. Afulani^9^; Bridgette Blebu^10^; Cinthia Blat^7^; Mary A. Garza^5^; Charles E. McCulloch^11^

^1^Department of Obstetrics, Gynecology and Reproductive Sciences, University of California, San Francisco, University of California San Francisco, CA; ^2^Department of Psychiatry and Behavioral Sciences, University of California, San Francisco, San Francisco, CA; ^3^Division of Maternal‐Fetal Medicine at the University of California San Francisco, University of California San Francisco, CA; ^4^Oregon Health & Science University‐Portland State University School of Public Health, Portland, OR; ^5^Central Valley Health Policy Institute at California State University, Fresno, Fresno, CA; ^6^Department of Obstetrics, Gynecology and Reproductive Sciences, University of California, San Francisco, UCSF, CA; ^7^Department of Obstetrics, Gynecology and Reproductive Sciences, University of California, San Francisco, San Francisco, CA; ^8^Department of Human Ecology, College of Agricultural and Environmental Sciences, University of California, Davis, Davis, CA; ^9^Department of Obstetrics, Gynecology and Reproductive Sciences, University of California, San Francisco, Universiy of California San Francisco, CA; ^10^Lundquist Institute for Biomedical Innovation ‐ Harbor‐UCLA, Los Angeles, CA; ^11^University of California, San Francisco, University of California San Francisco, CA

**Objective**: To compare the effect of two enhanced prenatal care models on perinatal depression and anxiety.

**Study Design**: Engaging Mothers and Babies – Reimagining Antenatal Care for Everyone (EMBRACE) is a pragmatic, randomized comparative‐effectiveness trial evaluating the effect of enhanced group prenatal care (eGPC) vs enhanced individual prenatal care (eIPC) on mental health (primary aim), care experiences (secondary aim), and preterm birth (exploratory aim) among Medicaid‐eligible people in California's San Joaquin Valley. In this analysis, we focused on changes in depression symptom severity (primary outcome, Patient Health Questionnaire‐9, scores range from 0–27) and anxiety symptom severity (secondary outcome, Generalized Anxiety Disorder‐7 scale, scores range from 0–21) from baseline (≤24 weeks gestation) to 3 months postpartum. We conducted primary analyses by intention‐to‐treat and subgroup analyses by prenatal care visit attendance (≥1 visit, ≥4 visits) and self‐reported race and ethnicity (Latine, Black). To estimate the intervention effect, we used linear mixed models that adjusted for baseline value of the outcome, self‐reported history of a mental health condition, and language of interview, with random intercepts for provider.

**Results**: 652 participants (*n* = 281 eGPC, n = 371 eIPC), enrolled in the study, of whom 72.2% identified as Latine and 7.4% identified as Black. Participants in both randomization groups experienced statistically significant small‐to‐moderate reductions in depression symptoms from baseline to three months postpartum. There were no significant differences in depression or anxiety symptom severity between the two enhanced prenatal care models, either in the full sample (intention‐to‐treat), by number of visits attended, or within the Latine or Black subgroups.

**Conclusion**: This study adds to the growing evidence that enhanced prenatal care, whether delivered in group or individual formats, can support meaningful improvements in perinatal mental health among low‐income populations.

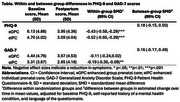

**1:15 PM – 1:30 PM**

## 10 | **Hemoglobin Equilibration After Transfusion in Postpartum Patients**

Ellen H. Crowe^1^; Ahmed Zaki Moustafa^2^; Megan C. Shepherd^3^; Baha M. Sibai^3^; Sean C. Blackwell^3^; Aaron W. Roberts^1^

^1^Department of Obstetrics, Gynecology and Reproductive Sciences, McGovern Medical School at UTHealth Houston, Houston, TX; ^2^Division of Maternal‐Fetal Medicine, Department of Obstetrics, Gynecology and Reproductive Sciences, McGovern Medical School at UTHealth Houston, University of Texas ‐ Houston, TX; ^3^Division of Maternal‐Fetal Medicine, Department of Obstetrics, Gynecology and Reproductive Sciences, McGovern Medical School at UTHealth Houston, Houston, TX

**Objective**: Common practice to determine patients’ response to uncomplicated blood transfusion is to assess hemoglobin concentration 4 hours post‐transfusion. The need for this practice has been disproven in non‐obstetric surgical patients, and patients with gastrointestinal bleeding. Given known fluid shifts that occur post‐delivery, it is still unknown whether postpartum patients require time to “equilibrate” to the true steady state hemoglobin value after completing transfusion. Our goal is to determine if waiting 4 hours following transfusion to reassess the hemoglobin level is necessary. If hemoglobin concentration equilibration occurs immediately post‐transfusion, delays in reassessment and management of postpartum anemia could be avoided.

**Study Design**: This is a prospective study in which serial hemoglobin assessments were completed following the transfusion of packed red blood cells (PRBCs) (immediate, 4 hours, 12 hours after transfusion). Patients were excluded if there was suspicion for ongoing bleeding, hemodynamic instability, planned return to the operating room, >72 hours from delivery, or if a collection timing was missed within 15 minutes of the protocol. Power analysis determined 23 patients would be needed for 80% power to detect a difference of 0.6g/dL using paired t‐tests with a *p* value of <0.05.

**Results**: From February 2025‐July 2025, a total of 38 patients were identified that received PRBC transfusion due to acute blood loss at delivery. 25 met criteria and completed the study protocol. There was no difference between mean hemoglobin concentration in g/dL between the immediate (0.16 g/dL, 95% CI −0.41 – 0.26) and 4‐hour post transfusion (0.14 g/dL, 95% CI −0.38 – 0.21) hemoglobin when compared to 12‐hour post transfusion hemoglobin (Figure 1).

**Conclusion**: An arbitrary 4‐hour delay between completion of blood transfusion and laboratory testing is not required for assessment in postpartum patients to determine steady state hemoglobin level. Earlier evaluation of hemoglobin following transfusion could more quickly determine the need for additional interventions or transfusion.

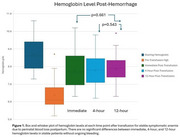

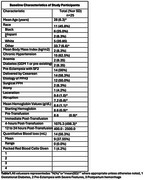

**1:30 PM – 1:45 PM**

## 11 | **Optimizing Iron Deficiency Management to Reduce Maternal Anemia and Peri‐Delivery Transfusion: A QI Initiative**

Carolyn M. Webster^1^; Charlotte B. McCarley^2^; Gabriella D. Cozzi‐Glaser^3^; Jori May^4^; Amy Boone^5^; Ashley N. Battarbee^6^

^1^University of Alabama at Birmingham, Birmingham, AL; ^2^University of Tennessee Medical Center, Knoxville, TN; ^3^Feinberg School of Medicine, Chicago, IL; ^4^University of Alabama at Birmingham, University of Alabama at Birmingham, AL; ^5^Indiana University, Indianapolis, IN; ^6^Center for Research in Women's Health, University of Alabama at Birmingham, Birmingham, AL

**Objective**: Iron deficiency (ID) is associated with anemia and adverse obstetrical outcomes. Iron supplementation is standard, but there is no consensus on optimal methods of repletion, and prior studies have failed to show improvement in obstetrical outcomes.

Due to high rates of maternal anemia and peri‐delivery transfusion at our tertiary care center, we developed a quality improvement (QI) algorithm for early identification and treatment of ID. We sought to evaluate the impact on anemia, transfusion, and other adverse outcomes.

**Study Design**: Observational cohort study of patients who delivered at ≥23 weeks GA before (1/2021–9/2023) and after (1/2024–3/2025) QI algorithm implementation, excluding a 3‐month washout and sickle cell disease. The algorithm included universal ferritin screening (intake and 24–28 wks) and treatment of ID with oral or IV iron based on anemia severity and GA. The primary outcome was anemia at delivery admission (Hgb <10.5 mg/dL in 2nd trimester; Hgb <11 mg/dL in 3rd trimester). Secondary outcomes were peri‐delivery transfusion, moderate and severe anemia, mean Hgb, and select infant outcomes. QI process measures included ferritin testing and IV iron therapy. Multivariable logistic regression estimated the association between post‐implementation and outcomes.

**Results**: Of 13,968 included patients: 9237 (66%) delivered pre‐implementation and 4731 (33%) post‐ implementation. Compliance was high: 83% of those initiating care <28 wks had ≥2 ferritin values. IV iron use increased >7‐fold (3% v 23%). Deliveries post‐implementation had lower odds of both pre‐delivery anemia (36% v 23%, aOR 0.48, CI 0.43–0.53) and transfusion (5.9% v 5.0%, aOR 0.71, CI 0.59–0.87). Neonatal outcomes were similar, although a small increase in mean birthweight was seen post‐implementation (*p* = 0.02; Table 2).

**Conclusion**: Early identification and treatment of maternal ID using a standardized algorithm with universal ferritin testing and iron repletion reduced pre‐delivery anemia by 50% and transfusion by 30% at our center. Further study is needed to evaluate generalizability and cost‐effectiveness of this approach.

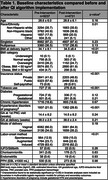

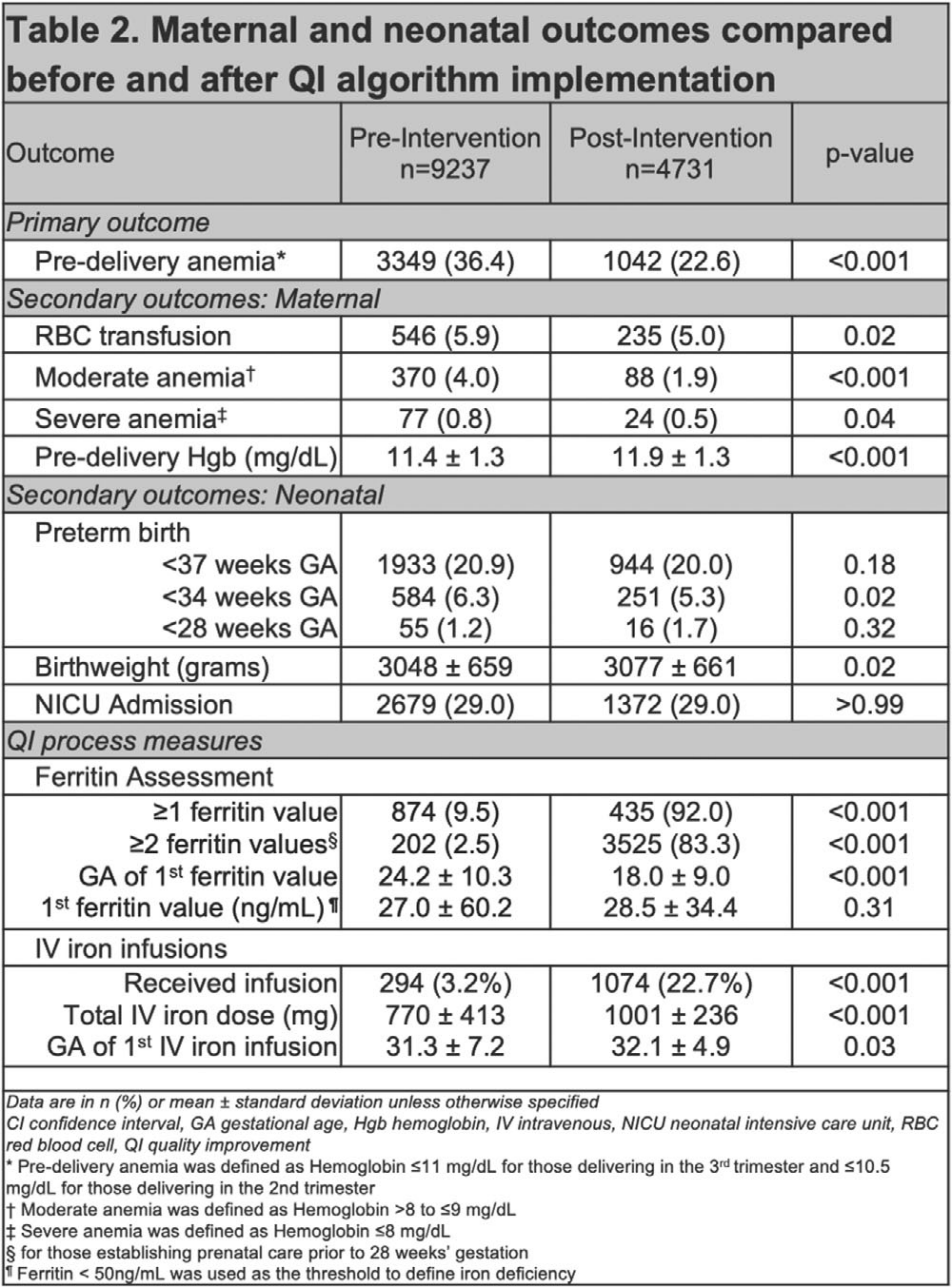

**1:45 PM – 2:00 PM**

## 12 | **Association Between Uterine Artery Doppler Velocimetry, Placenta Accreta Spectrum, and Degree of Placental Invasion**

Elias Kassir^1^; Edgar A. Hernandez‐Andrade^2^; Donatella Gerulewicz^2^; Sarah T. Mehl^2^; Eleazar E. Soto^1^; Ramesha Papanna^1^; Sean C. Blackwell^2^; Baha M. Sibai^2^; Farah H. Amro^2^

^1^University of Texas Health Science Center in Houston, McGovern Medical School, Houston, TX; ^2^Division of Maternal‐Fetal Medicine, Department of Obstetrics, Gynecology and Reproductive Sciences, McGovern Medical School at UTHealth Houston, Houston, TX

**Objective**: The increase in uterine vascularity in patients with placenta accreta spectrum (PAS) may be reflected in the uterine arteries(UtA). We aim to identify if there are differences in UtA Doppler indices in patients with and without PAS, and if these indices can predict PAS severity.

**Study Design**: This was a prospective cohort of 136 patients referred to an academic referral center for suspected PAS. Following referral, patients were deemed to either be low‐risk or high‐risk for PAS based on ultrasound imaging. At the time of referral, the left and right UtA were evaluated (∼1 cm from crossing with external iliac artery, wall filter 90Hz, angle of insonation close to 0), and mean values of the peak systolic velocity (PSV), end‐diastolic velocity (EDV), and pulsatility index (PI) were calculated. Mean values were converted to z‐scores to adjust for differences in gestational age. PAS diagnosis and severity were confirmed by pathology following hysterectomy. Comparisons were made to previously published reference values for the UtA in uncomplicated pregnancies. Differences in Doppler indices were analyzed using Student's t‐test and ANOVA with post‐hoc tests.

**Results**: Sixty‐three out of 136 (46.3%) patients did not have PAS. 73/136 (53.7%) patients had PAS and underwent hysterectomy. Of these, 17/73 (23.3%) had accreta, 40/73 (54.8%) had increta, 16/73 (21.9%) had percreta.

Mean PI was similar in patients with PAS compared to normal reference values; PSV and EDV were higher in patients with PAS. (Figure 1)

Mean z‐PI was not significantly different between patients with (mean 0.23, SD 0.9) or without PAS (mean 0.19, SD 1.02), *p* = 0.87. Mean z‐PSV and z‐EDV were significantly higher in women with PAS (PSV mean 7.05, SD 3.1; EDV mean 1.35, SD 3.4) compared to those without PAS (PSV mean 4.15, SD 2.9, *p* < 0.001; EDV mean 0.53, SD 1.4, *p* = 0.007). (Figure 2) No differences in z‐PI, z‐PSV, or z‐EDV were seen based on PAS severity.

**Conclusion**: In those with PAS, UtA Doppler indices are not different based on the degree of placental invasion. However, UtA PSV and EDV are higher in patients with PAS compared to those without PAS.

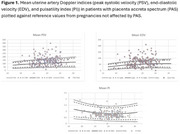

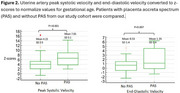

**2:00 PM – 2:15 PM**

## 13 | **Investigating the Relationship Between Prenatal Methadone Exposure and Neonatal Withdrawal Using Cord Tissue Analysis**

Courtney Townsel^1^; Makeda Turner^2^; Fatemeh Abdollah Biroon^2^; Vedavalli Govindan^3^; Eric K. Broni^4^; Jeannie C. Kelly^5^

^1^University of Maryland School of Medicine, University of Maryland School of Medicine, MD; ^2^University of Maryland School of Medicine, Baltimore, MD; ^3^Campbell University School of Osteopathic Medicine, Lillington, NC; ^4^Yale University, New Haven, CT; ^5^Washington University School of Medicine, Washington University School of Medicine, MO

**Objective**: Neonatal opioid withdrawal syndrome (NOWS) is more common among neonates exposed to methadone than buprenorphine prenatally. Despite this maternal dose of methadone does not predict severity of NOWS. We set out to assess whether umbilical cord methadone and metabolite levels are related to maternal methadone dose and NOWS severity.

**Study Design**: Secondary analysis of a multi‐site prospective study of pregnancies with opioid use disorder (OUD) between 2021–2025. Patients included if maintained on methadone, delivered ≥34 weeks and had biospecimen collected at delivery. Major fetal anomalies and missing data were exclusions.  Maternal and neonatal characteristics were abstracted. Umbilical cord tissue was collected after delivery, drained of blood, stored at −80°C and underwent mass spectrometry for quantification of methadone and 2‐ethylidene‐1,5‐dimethyl‐3,3‐diphenylpyrrolidine (EDDP) levels. NOWS was defined as any Finnegan score >4; severe NOWS as requiring pharmacotherapy or Finnegan score >24 in 24 hours. Spearman correlations assessed relationships between maternal dose, cord drug levels and NOWS. *p* < 0.05 was significant.

**Results**: Out of 83 patients with OUD, 49 were maintained on methadone. Patients were mostly non‐Hispanic White (92%), multiparous (67%) and underwent cesarean (57%). NOWS was diagnosed in 90% (*n* = 44) of neonates, with 57% (*n* = 28) having severe NOWS. Maternal methadone dose strongly correlated with umbilical cord methadone (*r* = 0.63, *p* < 0.001) and moderately correlated with umbilical cord EDDP (*r* = 0.33, *p* = 0.02). As maternal methadone dose increased so did cord methadone and EDDP levels (*p* < 0.001). Cord methadone and EDDP levels were not different between non‐severe and severe NOWS cases. Cord EDDP, but not cord methadone, levels show a significant positive correlation with Finnegan scoring (*r* = 0.38, *p* = 0.02).

**Conclusion**: Maternal methadone dose correlated with neonatal cord drug levels, but neither predicted NOWS severity. Cord EDDP levels showed a strong association with Finnegan scores and may be the best NOWS biomarker. NOWS severity likely involves factors beyond drug exposure.

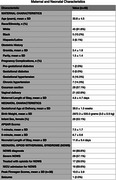

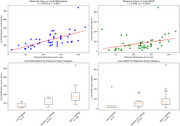

**2:15 PM – 2:30 PM**

## 14 | **Role of Iron Supplementation in Pregnant Women With Non‐Anemic Iron Deficiency: A Randomized Controlled Trial**

Bijal Parikh^1^; Mayi Gnofam^2^; Rakasa Pattanaik^3^; Brynn S. Franz^4^; Tiffany Yang^5^; Emily Stetler^2^; Chaitali Korgaonkar‐cherala^6^; Heidi Preis^5^; David J. Garry^7^; Cassandra Heiselman^8^; Kimberly Herrera^8^

^1^St. Peters University Hospital, Warren, NJ; ^2^Stony Brook University, New York, NY; ^3^Beth Israel Deaconess Medical Center, Boston, MA; ^4^University of South Carolina School of Medicine ‐ ‐ Columbia, SC, East Northport, NY; ^5^Stony Brook University, Stony Brook, NY; ^6^Stony Brook Medicine, Stony Brook, NY; ^7^Stony Brook Medicine, Stony Brook Medicine, NY; ^8^Stony Brook University Hospital, Stony Brook, NY

**Objective**: Non‐anemic iron deficiency (NAID) in pregnancy is common and a known risk factor for the development of anemia. Our study aimed to assess whether treatment of earlier pregnancy NAID decreased the progression to anemia at delivery.

**Study Design**: This was a double‐blinded, randomized controlled trial at a single academic center from 08/2022 to 03/2024. Patients with NAID, defined as low ferritin (<30 mcg/L) and normal hemoglobin (<11 g/dL) were included. Patients were excluded if they had significant vaginal bleeding, active iron supplementation, iron hypersensitivity, or chronic illness. Participants were randomized to receive an iron supplement or placebo pill daily and prenatal care providers were blinded to treatment arm. The primary outcome was incidence of anemia at the time of admission, defined as Hgb <11 g/dL. Intention‐to‐treat analysis included chi‐square tests, t‐tests or nonparametric equivalents, and regression modeling.

**Results**: There were 132 patients with NAID included in the study with 64 in the treatment group and 68 in the control group. No significant differences in baseline characteristics were observed between the groups except for lower first trimester ferritin levels in the treatment group similar, and no differences were observed in attrition (Table 1). There was no significant difference between Hgb levels (11.8 ± 1.4 vs 11.6 ± 1.3, *p* = 0.26) or anemia on admission across treatment versus controls (28.3% vs 29.9%, *p* = 0.85) Higher BMI at first prenatal visit (*p* = 0.014) was associated with lower rates of admission hemoglobin. In the multivariable regression for admission anemia, treatment group membership was still not significant. Rates of antepartum oral iron prescription were higher among the controls (29.4%) compared to the treatment group (14.1%) (*p* = 0.03). There were no differences in delivery and neonatal outcomes between groups (Table 2).

**Conclusion**: There is no difference between groups in the rate of anemia at that time of admission; however, the treatment group is less likely to require additional oral iron supplementation.

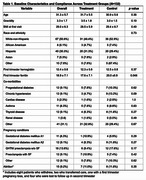

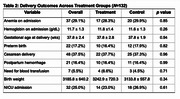

**2:30 PM – 2:45 PM**

## 15 | **Goal Maternal Hemoglobin in 2nd Trimester Following Iron Therapy to Achieve Non‐Anemic State by Delivery**

Rupsa C. Boelig^1^; Mrutyunjaya Bellad^2^; Manjunath Somannavar^2^; Simal Thind^3^; Michael Georgieff^4^; Sudhir Bhandari^5^; Sudhir Mehta^5^; Seema Mehta^5^; Dharmesh Kumar Sharm^5^; Yogesh Kumar^2^; Umesh Charantimath^2^; Amaresh Patil^2^; Ashalata Mallapur^6^; Umesh Ramadurg^6^; Radha Sangavi^7^; Praveen Patil^7^; Subarana Roy^8^; Phaniraj Vastrad^8^; Chander Shekhar^3^; Benjamin Leiby^3^; Rebecca Hartmann^3^; Richard Derman^3^

^1^Sidney Kimmel Medical College, Thomas Jefferson University, Philadelphia, PA; ^2^KLE Academy of Higher Education and Research J N Medical College, Belagavi, Karnataka; ^3^Thomas Jefferson University, Philadelphia, PA; ^4^University of Minnesota, Minneapolis, MN; ^5^Sawai Man Singh Medical College, Jaipur, Rajasthan; ^6^S Nijalingappa Medical and HSK Hospital and Research Center, Bagalkote, Karnataka; ^7^Raichur Institute of Medical Sciences, Raichur, Karnataka; ^8^Model Rural Health Research Unit, Sirwar, Karnataka

**Objective**: In a recent multicenter randomized controlled trial in India comparing intravenous to oral iron for early intervention of maternal anemia, ∼30% of participants in all arms failed to achieve Hb ≥11.0 g/dL by delivery. We aim to describe the natural history of hemoglobin progression in patients with moderate iron deficiency anemia (IDA) following initial therapy to identify goal Hb necessary to maintain non‐anemic state through the end of pregnancy without additional interventions.

**Study Design**: This is a secondary analysis of a multi‐center randomized controlled trial in India comparing single dose intravenous (IV) iron to oral iron for moderate IDA (Hb 7.0–9.9 g/dL) at 14–17 weeks gestation. We excluded those with preterm birth, missing values, or any additional iron therapy/transfusion prior to delivery. The primary outcome for this analysis was maternal Hb ≥ 11.0 g/dL by delivery. We evaluated prediction of Hb at delivery by 1) Regression line to identify Hb at 20–24 weeks predictive of Hb ≥ 11.0 g/dL and 2) Receiver operating characteristic curve (ROC) and Youden index predictive of achieving ≥11.0 g/dL

**Results**: 3021 of 4368 in trial were included: 866, 1100, and 1055 in oral iron, IV ferric derisomaltose, and IV ferric carboxy maltose arms respectively. More in oral group (40%) were excluded compared to IV groups (24% and 28% respectively). The Hb trend for both treatment groups in Figure 1, with distinct patterns in those who achieve Hb ≥ 11.0 by delivery vs not. In terms of threshold Hb at 20–24 weeks, based on the regression line the threshold Hb is ≥ 9.68 (9.52–9.84) for oral and Hb ≥ 10.04 (9.90–10.18) for IV (Figure 2). Using ROC analysis, the cutpoints are Hb ≥ 10.29 for oral and Hb ≥ 10.58 for IV. The PPV is similar for treatment groups (oral 78%, IV 74%).

**Conclusion**: For pregnancies complicated by IDA treated at 14–17 weeks, achieving Hb of 10.5 g/dL by 20–24 weeks can identify those likely to have Hb ≥ 11.0 at delivery without additional interventions. This benchmark can be used to identify those who would benefit from additional iron therapy earlier in pregnancy to achieve non‐anemic state by delivery.

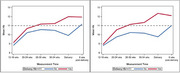

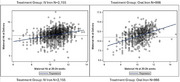

**2:45 PM – 3:00 PM**

## 16 | **Contemporary Patterns and Outcomes of Antidepressant Discontinuation in Pregnancy**

Kelly B. Zafman^1^; Yifan Zhu^2^; Sara Kornfield^3^; Aaron Smith‐McLallen^2^; Sindhu K. Srinivas^4^

^1^University of Pennsylvania, Philadelphia, PA; ^2^Independence Blue Cross, Philadelphia, PA; ^3^Hospital of the University of Pennsylvania, Hospital of the University of Pennsylvania, PA; ^4^Department of Obstetrics and Gynecology, Perelman School of Medicine, Maternal and Child Health Research Center, Philadelphia, PA

**Objective**: Mental health disorders are the largest contributor to maternal mortality, yet there are many pressures to stop antidepressants in pregnancy, including the recent FDA panel. Our objective was to evaluate contemporary patterns and outcomes of antidepressant discontinuation in pregnancy in patients with mental health disorders.

**Study Design**: This was a cross‐sectional analysis of a state‐based private insurance database. Diagnosis Related Groups and ICD‐10 codes were used to identify patients who delivered Jan 1^st^, 2023‐Dec 31^st^, 2024, with a diagnosis of depression/anxiety prior to pregnancy. Patients with an active prescription for a selective serotonin reuptake inhibitor (SSRI) or serotonin norepinephrine reuptake inhibitor (SNRI) 3 months prior to pregnancy were included. Pharmacy claims data was used to ascertain SSRI/SNRI use in pregnancy. Treatment discontinuation was defined as no fills in pregnancy or gap >60 days. We compared differences among patients who continued and discontinued antidepressants.

**Results**: Of 3983 patients with depression/anxiety, 1462 (36.7%) entered pregnancy with an active SSRI/SNRI. 260 (17.8%) patients had no medication fills in pregnancy; 945 (64.6%) had a >60‐day gap. Similar rates of discontinuation were seen across trimesters, with 29.7%, 31.6%, and 38.6% per trimester. Patients who continued and discontinued antidepressants had similar rates of outpatient and emergency visits for mental health indications prior to pregnancy. However, patients who discontinued medications were more likely to have a mental health emergency during pregnancy with peaks in the 1st (58/1000 vs. 37/1000, *p* = 0.02) and 9th month of pregnancy (59/1000 vs. 29/1000, *p* < 0.01).

**Conclusion**: Treatment for mental health conditions should not be withheld during pregnancy.  Despite this recommendation, the majority of patients discontinued SSRI/SNRI therapy which is associated with an almost two‐fold higher risk of mental health emergencies.  Strategies to promote treatment continuation in pregnancy should be an urgent public health priority as a strategy to mitigate the maternal mortality crisis.

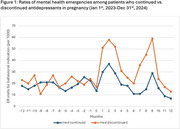

**3:00 PM – 3:15 PM**

## 17 | **Engagement Drives Detection: Rethinking Postpartum Behavioral Health Screening**

Miriam J. Alvarez^1^; Anna Madden‐Rusnak^1^; Colby Brown^1^; Alejandro Guevara^2^; Vaishnavi V. Kankate^1^; Lorie M. Harper^1^; Nandini Raghuraman^3^; Erin Richardson^4^; Jeffrey Newport^5^; George A. A. Macones^1^; Alison G. G. Cahill^6^

^1^Department of Women's Health, Dell Medical School at the University of Texas at Austin, Austin, TX; ^2^Texas Tech University Health Sciences Center Paul L. Foster School of Medicine, El Paso, TX; ^3^Washington University School of Medicine, Washington University School of Medicine, MO; ^4^Women's Reproductive Mental Health, Dell Medical School at the University of Texas at Austin, Austin, TX; ^5^Women's Reproductive Mental Health, Dell Medical School at the University of Texas at Austin, Austin, TX; ^6^Department of Women's Health, Dell Medical School at the University of Texas at Austin, Department of Women's Health, Dell Medical School at the University of Texas at Austin, TX

**Objective**: Postpartum behavioral health (BH) symptoms disproportionately affect Hispanic and Spanish‐speaking women, yet barriers to screening and engagement remain. This study evaluated predictors of BH symptom detection to inform care strategies for this underserved population.

**Study Design**: In this prospective cohort study, 262 women were enrolled at delivery into one of four postpartum (PP) screening protocols (Table): Standard Care (baseline only), Moderate (baseline + every 4 weeks through 24 weeks PP), High (baseline + every 2–4 weeks through 24 weeks PP), or Intensive (biweekly through 36 weeks PP + two return‐to‐work screeners). Women returning to work within 6 months were assigned to Intensive; others were randomized. Primary outcomes included depression, anxiety, PTSD, and a composite BH outcome (positive on any). Engagement was defined by completion rate (≥85% = fully, <85% = partially). Chi‐square and Fisher's exact tests were used for comparisons; logistic regression (excluding Standard Care) evaluated associations with group, engagement, return‐to‐work, and birth complications.

**Results**: Of 262 women, 13% (*n* = 34) screened positive for at least one BH condition. Detection rates did not significantly differ by screening protocol (composite: 12%–16%, *p* = 0.91). Depression rates were similar across groups (9%–12%, *p* = 0.91), while anxiety and PTSD varied but were not significant (anxiety: 2%–9%, *p* = 0.35; PTSD: 0%–3%, *p* = 0.85). Regression showed no association between group, return‐to‐work, or birth complications and the composite BH outcome (*p* > 0.05). However, engagement was a strong predictor: partially engaged participants had significantly higher odds of screening positive (aOR = 6.9; 95% CI: 2.1–22.4; *p* = 0.01).

**Conclusion**: Among Hispanic, Spanish‐speaking PP women, screening engagement—not frequency—was the strongest predictor of BH symptom detection. These findings suggest that partially engaged women may represent a systematically higher‐risk group. Identifying and developing strategies to reach those with inconsistent engagement is critical to improving BH outcomes in this population.

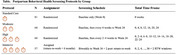

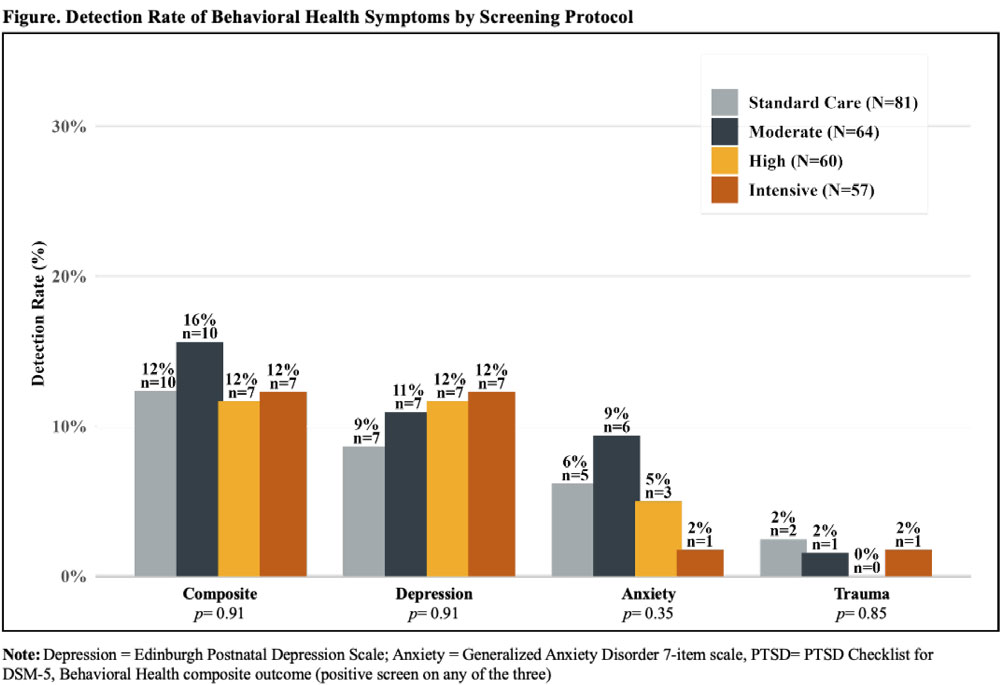

**3:15 PM – 3:30 PM**

## 18 | **Placental Immune Cell and Oxygenation Profiles in Opioid‐Exposed Pregnancies: A Longitudinal MRI Study**

Jeannie C. Kelly^1^; Zezhen Xiang^1^; Sam A. S. Williams^1^; Melissa Mills^2^; Emily Diveley, BSN^1^; Nandini Raghuraman^1^; Peinan Zhao^1^; Nardhy Gomez‐Lopez^1^; Antonina I. Frolova^3^; Yong Wang^1^

^1^Washington University School of Medicine, Washington University School of Medicine, MO; ^2^Washington University School of Medicine, Saint Louis, MO; ^3^Washington University School of Medicine, St. Louis, MO

**Objective**: Prenatal opioid exposure (POE) is linked to adverse perinatal outcomes and impaired neurodevelopment. Animal models have demonstrated opioid‐induced neuroinflammation; however, effects on the human placenta remain poorly defined. We hypothesized that POE disrupts both placental immune response and oxygenation. To test this hypothesis, we compared immune and oxygenation profiles between opioid‐exposed and unexposed pregnancies using longitudinal MRI.

**Study Design**: Pregnant patients with chronic POE were prospectively recruited and matched 1:1 (fetal sex, maternal race, Area Deprivation Index) to unexposed controls confirmed by urine screens. Participants underwent up to one MRI per trimester (12–36 weeks). Immune cell infiltration (“cell ratio”) was assessed using a previously validated imaging method developed by our team (Figure A); T2* mapping assessed oxygenation. A fuzzy clustering algorithm segmented placental tissue, vessels, and intervillous space using T2*, apparent diffusion coefficient, and fractional anisotropy. Differences were assessed with Wilcoxon Rank‐Sum and likelihood ratio tests.

**Results**: 20 participants (10 POE; 10 controls) with similar demographics were included (Table). Longitudinal analysis showed a progressive increase in immune cell infiltration from the first to the third trimester in POE cases, which was not observed in controls (Figure B). Consistently, immune cell ratios in the third trimester were higher in POE cases than in controls (Figure C). Although overall mean T2* values did not differ between groups (54.4 ± 16.9 vs. 71.3 ± 9.6 ms; *p* =  0.095), longitudinal analysis revealed persistently lower T2* values in POE placental tissue (F = 11.04, *p* =  0.0005; Figure D), with no differences in placental vessels or intervillous space.

**Conclusion**: POE reshapes the placental environment, with increased immune cell infiltration in the third trimester and reduced placental tissue oxygenation throughout gestation. This study provides the first noninvasive evidence of POE‐induced placental immune pathology, suggesting a mechanism for adverse outcomes and a target for future interventions.